# Increased metal content in the TDP-43^A315T^ transgenic mouse model of frontotemporal lobar degeneration and amyotrophic lateral sclerosis

**DOI:** 10.3389/fnagi.2014.00015

**Published:** 2014-02-11

**Authors:** Theresa N. T. Dang, Nastasia K. H. Lim, Alexandra Grubman, Qiao-Xin Li, Irene Volitakis, Anthony R. White, Peter J. Crouch

**Affiliations:** ^1^Department of Pathology, The University of MelbourneVIC, Australia; ^2^Florey Institute of Neuroscience and Mental Health, The University of MelbourneVIC, Australia

**Keywords:** amyotrophic lateral sclerosis (ALS), frontotemporal lobar degeneration (FTLD), TAR DNA binding protein-43 (TDP-43), copper (Cu), zinc (Zn), manganese (Mn), neurodegenerative disease

## Abstract

Disrupted metal homeostasis is a consistent feature of neurodegenerative disease in humans and is recapitulated in mouse models of Alzheimer’s disease, Parkinson’s disease, amyotrophic lateral sclerosis (ALS) and neuronal ceriod lipofuscinosis. While the definitive pathogenesis of neurodegenerative disease in humans remains to be fully elucidated, disease-like symptoms in the mouse models are all driven by the presence or over-expression of a putative pathogenic protein, indicating an *in vivo* relationship between expression of these proteins, disrupted metal homeostasis and the symptoms of neuronal failure. Recently it was established that mutant TAR DNA binding protein-43 (TDP-43) is associated with the development of frontotemporal lobar degeneration and ALS. Subsequent development of transgenic mice that express human TDP-43 carrying the disease-causing A315T mutation has provided new opportunity to study the underlying mechanisms of TDP-43-related neurodegenerative disease. We assessed the cognitive and locomotive phenotype of TDP-43^ A315T^ mice and their wild-type littermates and also assessed bulk metal content of brain and spinal cord tissues. Metal levels in the brain were not affected by the expression of mutant TDP-43, but zinc, copper, and manganese levels were all increased in the spinal cords of TDP-43^ A315T^ mice when compared to wild-type littermates. Performance of the TDP-43^ A315T^ mice in the Y-maze test for cognitive function was not significantly different to wild-type mice. By contrast, performance of the TDP-43^ A315T^ in the rotarod test for locomotive function was consistently worse than wild-type mice. These preliminary *in vivo* data are the first to show that expression of a disease-causing form of TDP-43 is sufficient to disrupt metal ion homeostasis in the central nervous system. Disrupted metal ion homeostasis in the spinal cord but not the brain may explain why the TDP-43^ A315T^ mice show symptoms of locomotive decline and not cognitive decline.

## INTRODUCTION

Metals such as iron, copper and zinc are needed for normal cell function and alterations to the bio-availability of these metals can therefore have devastating consequences on the survival and functionality of cells in all parts of the body. This is particularly true for functionality of the central nervous system (CNS) because metal ions are also required for normal synaptic function (Tamano and Takeda, 2011). Underscoring the significance of maintaining metal homeostasis in the CNS, disrupted metal homeostasis is evident in many neurodegenerative diseases including Alzheimer’s disease (AD), Parkinson’s disease (PD), and amyotrophic lateral sclerosis (ALS). In AD, iron, copper, and zinc accumulate within amyloid plaques (Connor et al., 1992; Lovell et al., 1998), in PD iron is increased within the substantia nigra (Sofic et al., 1988), and in ALS copper, zinc, manganese, and several other metals are all increased in the cerebrospinal fluid (Roos et al., 2013).

In addition to human tissue affected by neurodegenerative disease, animal models of these diseases also recapitulate elements of metal dyshomeostasis; AD model mice that over-express the amyloid-β precursor protein have decreased levels of copper and zinc in the brain relative to wild-type controls (Maynard et al., 2002) copper and iron are altered in the brains of PD model mice (Matusch et al., 2010; Ayton et al., 2013) and zinc and copper levels are altered in mutant SOD1 mouse models of ALS (Kiaei et al., 2004; Tokuda et al., 2013). The presence of disrupted metal homeostasis in animal models of neurodegenerative disease as well as the human condition has encouraged development of therapeutic strategies that aim to correct this imbalance (Badrick and Jones, 2011; Crouch and Barnham, 2012). However, confirming whether the loss of metal homeostasis in neurodegenerative disease represents a cause of the disease or a consequence of neuronal dysfunction still requires further investigation.

Frontotemporal lobar degeneration (FTLD) is a collective term for neurodegenerative diseases that involve degeneration of the frontal and temporal lobes of the brain (Pickering-Brown, 2010). It can affect individuals from the age of 40, and in the aged population, is one of the most common causes of dementia (Knopman et al., 2004). There are three main sub-types of FTLD based on their histology; dementia lacking distinct histology, FTLD with tau-positive inclusions, and FTLD with tau-negative ubiquitin-positive inclusions (FTLD-U). In 2006 it was established that cytoplasmic inclusions present in the brains of people with FTLD-U and in the spinal cords of people with ALS contained aggregates of the TAR DNA binding protein-43 (TDP-43) (Neumann et al., 2006). This provided the first molecular evidence to explain the comorbidity that is common to ALS and FTLD (Lomen-Hoerth et al., 2002) and also led several groups to propose that ALS and FTLD-U represent two ends of a spectrum of TDP-43-mediated neurodegenerative diseases that can collectively be referred to as TDP-43 proteinopathies (Liscic et al., 2008; Chen-Plotkin et al., 2010).

It is currently unclear whether disrupted metal homeostasis is present in TDP-43 proteinopathies. *In vitro* studies have implicated a role for metals in the aggregation of endogenous TDP-43 (Caragounis et al., 2010), and metal-based therapeutic strategies have the potential to prevent aberrant TDP-43 metabolism (Parker et al., 2012); however, there is a current paucity of *in vivo* data. This study utilizes the recently developed TDP-43^A315T^ mouse model of FTLD/ALS (Wegorzewska et al., 2009) to assess whether the presence of a pathogenic form of TDP-43 affects metal ion homeostasis *in vivo*, and if present, to assess whether altered metal homeostasis may be associated with the symptoms of neuronal decline in an animal model of TDP-43 proteinopathy.

## MATERIALS AND METHODS

### MATERIALS

Rabbit antibody to TDP-43 was purchased from Proteintech (USA); rabbit antibodies to FLAG, histone H3, and GAPDH were obtained from Cell Signaling (Australia). All other chemicals were purchased from Sigma Aldrich (Australia) unless otherwise stated.

### ANIMALS

TDP-43^A315T^ mice were purchased from The Jackson Laboratory Repository (Stock no. 010700; Bar Harbor, ME, USA). This mouse model was generated using the mouse prion promoter and a cDNA encoding human *TARDBP* with an A315T mutation and containing an N-terminal FLAG-tag (Wegorzewska et al., 2009). A colony of TDP-43^A315T^ mice was maintained by breeding TDP-43^A315T^ mice with non-transgenic C57/BL6 mice. Animals were group-housed under standard housing conditions with a 12 h light–dark cycle, and food and water *ad libitum*. All animals expressing the TDP-43^A315T^ were confirmed via PCR according to the distributor’s protocol. Non-transgenic littermates not expressing the TDP-43^A315T^ were used as wild-type controls. All animal protocols and procedures were approved by Melbourne Research Animal Ethics at The University of Melbourne, Australia. Only male mice were included in this study and the number of animals per group is stated in the figure legends.

### BEHAVIORAL TESTING

Locomotor activity was assessed using the rotarod assay (Turner et al., 2009); rod diameter 35 mm and elevation 200 mm. Mice were acclimatized to the rotarod by daily training for one week. Training involved three 5-min training runs per mouse per day. For each training run the mice were returned to the rotarod if they fell before the end of the 5-min period. Once the mice reached 5 weeks of age they were tested twice weekly. During testing, the rotarod was set to accelerate from 4 to 40 rpm over 5 min. Latency to fall was recorded in seconds. On each testing day each mouse was tested twice and only higher latency to fall score used for analysis.

Cognitive function was assessed using the Y-maze test as previously described (Hung et al., 2012) and performed at the age of 10 weeks. The floor of the maze was covered with sawdust which was replaced between each training and testing run in order to prevent residual odors that may affect performance. The walls of the maze were non-transparent and decorated internally so that each arm was visually unique. For training each mouse was allowed to explore only two arms of the maze (the novel arm was blocked with a non-transparent wall) for 10 min. At the end of training mice were removed from the maze for a period of 1 h before returning and being allowed to explore all 3 arms of the maze for 5 min. For training and testing the mice were placed into the maze at the same position in the starting arm of the maze. During testing the number of entries made to each arm of the maze was recorded and the percentage of entries into the novel third arm was calculated.

### TISSUE COLLECTION AND PREPARATION

Male mice (mean age of 12 weeks) were anesthetized by intraperitoneal injection of PBS (137 mM NaCl, 8.1 mM Na_2_HPO_4_, 2.68 mM KCl, 1.47 mM KH_2_PO_4_, pH 7.4) supplemented with ketamine (20 mg/mL) and xylazine (4 mg/mL). Animals were then transcardially perfused with PBS containing 0.25% phosphatase inhibitor cocktail, 1% protease inhibitor (Roche) and 20 U/mL heparin. Perfused brain, spinal cord, liver, and quadriceps muscle were collected, frozen on dry ice and stored at -80°C until further processing.

For western blot analysis, enzyme-linked immunosorbent assay (ELISA) and protein oxidation detection, tissues were mechanically homogenized in 150–200 μL homogenizing buffer [PBS containing 1:100 protease inhibitor cocktail (Roche), 1:50 phosphatase inhibitor cocktail and 1:20 DNase (Roche)] before ultrasonication at 2 amp for 10 s. Homogenates were centrifuged at 18,000 *g* for 3 min at 4°C and the supernatant (soluble fraction) collected and the pellet (insoluble fraction) retained. Protein content was determined by the bicinchoninic acid assay (Thermo Scientific, USA). Fractions were stored at -80°C until further analysis.

### WESTERN BLOT ANALYSIS

Soluble tissue fractions were prepared in 4× loading buffer [250 mM Tris, 20% (v/v) glycerol, 8% (w/v) SDS, 2% (v/v) β-mercaptoethanol, 0.01% (w/v) bromophenol blue] and heated for 5 min at 95°C. Samples containing 10–30 μg protein were then loaded onto 4–12% NuPAGE Novex Bis–Tris Midi gels (Life Technologies) electrophoresed at 200 V for 40 min. Proteins were transferred onto PVDF membranes using the iBlot gel transfer device (Life Technologies) for 7 min according to the manufacturer’s instructions. Membranes were blocked with 4% (w/v) skim milk in PBS containing 0.05% (v/v) Tween-20 (PBST) followed by incubation with primary antibody (1:1000) overnight at 4°C. After washes in PBST, membranes were incubated with secondary antibody for 1 h at room temperature. Membranes were incubated with Western Lighting Ultra ECL (Perkin-Elmer) and imaged using the Fujifilm LAS-3000 Image reader. Blots were then stripped with 1% HCl for 15 min and reprobed for GAPDH or histone H3 as loading control. The optical density (OD) of bands was quantified using the ImageJ software and standardized to loading control (GAPDH/histone H3). The relative fold change for proteins in TDP-43^A315T^ mouse tissue is expressed relative to wild-type mouse tissue.

### METAL ANALYSIS

Inductively coupled plasma mass spectrometry (ICP-MS) was used to measure bulk metal concentration in tissue samples as described elsewhere (Maynard et al., 2002). Briefly, perfused brain and spinal cord tissue samples were lyophilized then digested in HNO_3_ (65% Suprapur, Merck) overnight. Liver and quadriceps muscles were used as control non-CNS tissue. Tissues were heated at 90^o^C before the addition of H_2_O_2_ (30% Aristar, BDH). Samples were left to stand for ~30 min, before further heating at 70^o^C. The average reduced volume was determined and samples were further diluted with 1% HNO_3_. Measurements were made using an Agilent 7700 series ICP-MS instrument using a Helium Reaction Gas Cell and 200 ppb of Yttrium (Y89) as an internal control (ICP-MS-IS-MIX1-1, Accustandard). Results are expressed as micrograms of metal per gram of wet weight tissue (μg/g).

### PROTEIN OXIDATION DETECTION AND MCP-1 ELISA

Oxidative stress and inflammation are associated with metal dyshomeostasis and neurodegeneration (Barnham et al., 2004; Molina-Holgado et al., 2007). To assess the oxidative and inflammatory status of the brain and spinal cord of the TDP-43^A315T^ we measured levels of oxidized proteins and the inflammation marker monocyte chemoattractant protein 1 (MCP-1). Brain and spinal cord soluble and insoluble fractions were analyzed for oxidative modified proteins via the OxyBlot Protein Oxidation Detection Kit (Merck Millipore, Australia) according to the manufacturer’s instructions. The OxyBlot detects the carbonyl groups found on oxidized proteins. The OD of bands was quantified using the ImageJ software and values were standardized to the loading controls GAPDH and histone H3 for soluble and insoluble fractions, respectively. The relative fold change of oxidized proteins in TDP-43^A315T^ mice is expressed relative to wild-type.**Levels of MCP-1 were measured in brain, spinal cord, liver and quadriceps muscle using the Mouse CCL2/JE/MCP-1 DuoSet (R&D Systems, USA) according to the manufacturer’s instructions.

### STATISTICAL ANALYSIS

All values are presented as mean ± SEM. All statistical analyses were performed using Graphpad Prism. Planned comparisons using two-tailed independent *t*-tests were used to analyze all data. Significance was set at *p *< 0.05.

## RESULTS

### TDP-43^A315T^ MICE EXHIBIT LOCOMOTOR IMPAIRMENT BUT NO COGNITIVE DEFICIT

**FIGURE 1:**
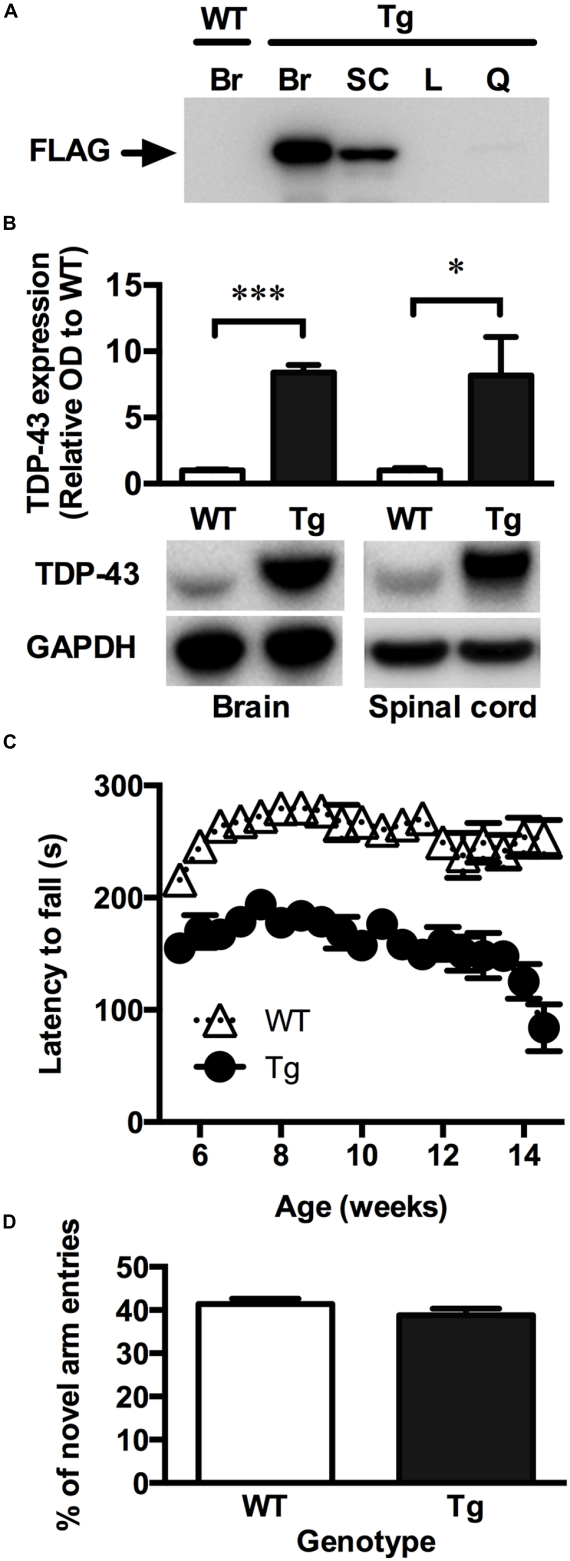
**TDP-43^A315T^ mice express mutant TDP-43 in the CNS and exhibit a locomotor impairment. (A)** Representative western blot showing the absence of mutant FLAG-tagged TDP-43 (FLAG) expression in the brain of wild-type (WT) mice and the expression of FLAG-tagged TDP-43 in the brain (Br), and spinal cord (SC) but not liver (L) and quadriceps muscle (Q) of transgenic TDP-43^A315T^ (Tg) mice. **(B)** Western blot analysis show a significant overexpression of total TDP-43 in the brain and spinal cord of Tg mice (*n *= 9) compared to WT (*n *= 9). ***p < 0.001; *p < 0.05. Blots shown are representative images. OD of bands were standardized to GAPDH and expressed relative to WT. **(C)** Tg mice exhibit a locomotor impairment compared to WT, scoring lower on the rotarod test than WT with a further decline in locomotor activity beginning at ~12 weeks. **(D)** No significant differences were detected in the number of novel arm entries of the Y-maze test between WT and Tg mice (*p *> 0.05).

Consistent with the original description of the TDP-43^A315T^ mice (Wegorzewska et al., 2009), the FLAG-tagged TDP-43^A315T^ was readily detected in the brain and spinal cord relative to the liver and quadriceps muscles, indicating that the mutant TDP-43 is mainly expressed within the CNS in this model (**Figure 1A**). When assessed using an antibody that detected both the FLAG-tagged mutant TDP-43 and the endogenous mouse TDP-43, expression of TDP-43 was ~7-fold higher in the transgenic animals compared to wild-type controls (**Figure 1B**).

To investigate whether the TDP-43^A315T^ mouse model recapitulate symptoms of ALS, the rotarod was used to assess locomotor activity. Lower scores on the rotarod are indicative of impaired locomotor function. TDP-43^A315T^ mice consistently scored lower than wild-type littermates at all time-points examined (**Figure 1C**). The poor performance of TDP-43^A315T^ mice on the rotarod decreased further towards the end of the study period, dropping sharply at ~12 weeks of age until end-stage at 14.2 ± 1.3 weeks. Unlike mutant SOD1 mouse models of ALS (Gurney et al., 1994; Wong et al., 1995) full hind-limb paralysis was not evident at end-stage in the TDP-43^A315T^ mice. Accordingly, the TDP-43^A315T^ mice maintained locomotor functionality through to end-stage (**Figure 1C**).

The Y-maze was used to examine whether the TDP-43^A315T^ mice exhibited a cognitive deficit reminiscent of FTLD. Cognitive function was examined in males at 10 weeks of age to ensure that testing was assessed before the dramatic late stage decline in locomotor function. The number of entries into the novel arm of the Y-maze, indicative of short-term memory function, did not differ significantly between the TDP-43^A315T^ mice and their age-matched wild-type littermates (*p *> 0.05; **Figure 1D**).

### INCREASED METAL LEVELS IN THE SPINAL CORD OF TDP-43^A315T^ MICE

**FIGURE 2:**
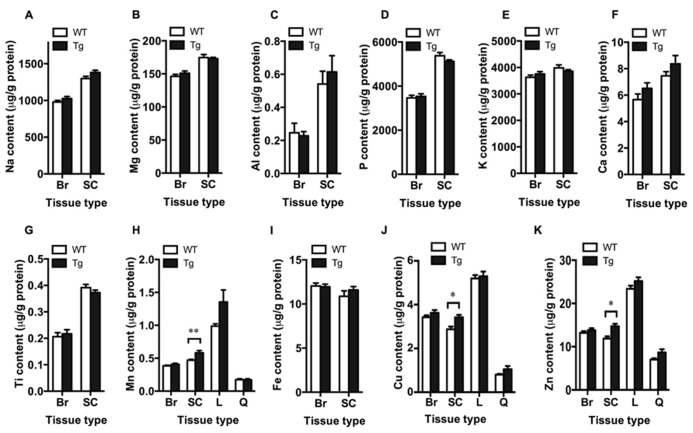
**Increased metal content in the spinal cord of TDP-43^A315T^ mice**. ICP-MS analysis of bulk metal content in the brain (Br), spinal cord (SC), liver (L) and quadriceps muscle (Q) of TDP-43^A315T^ mice (Tg), and wild-type litter mates (WT). **(A–K)** Planned comparisons revealed no significant differences in bulk levels of Na, Mg, Al, P, K, Ca, Ti, Mn, Fe, Cu, and Zn in the brain between genotyptes (*n *= 16 per genotype); however, **(H)** Mn, **(J)** Cu, and **(K)** Zn were significantly increased in the spinal cord of Tg mice compared to WT controls (*n *= 7 per genotype). **p < 0.01; *p < 0.5.

Bulk metal analysis was performed using ICP-MS and levels of Na, Mg, Al, P, K, Ca, Ti, Mn, Fe, Cu, and Zn were quantified in the brain and spinal cord of TDP-43^A315T^ and wild-type mice (**Figure 2**). Bulk levels of these metals were not significantly different in the brain between genotypes (*p* > 0.05; **Figures 2A–K**). However, Mn, Cu, and Zn were significantly increased in the spinal cord of TDP-43^A315T^ mice when compared to wild-type controls by 19.8, 16.9, and 18.8%, respectively (*p* < 0.05; **Figures 2H,J,K**). Changes to Mn, Cu, and Zn observed in the spinal cord tissue appeared to be restricted to this tissue; as per the brain, these metals were not altered in the liver or quadriceps muscle (**Figures 2H,J,K**).

### INCREASED OXIDATIVE STRESS IN THE SPINAL CORD OF TDP-43^A315T^ MICE

**FIGURE 3:**
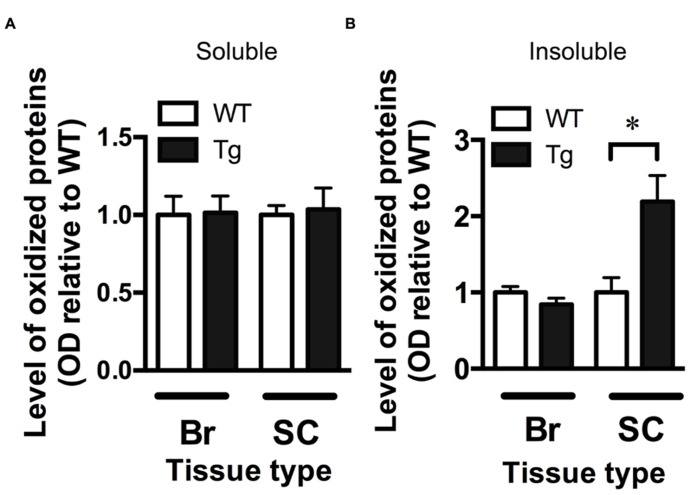
**Increased oxidative stress in the spinal cord of TDP-43^A315T^mice. (A)** The level of oxidized proteins as determined by OxyBlot is unchanged in the soluble fraction (containing cytosolic proteins) of the brain (Br) and spinal cord (SC) of TDP-43^A315T^ mice (Tg) compared to wild-type littermates (WT). **(B)** Levels of oxidized proteins were significantly increased in spinal cord insoluble fraction (containing membrane and nuclear material) of Tg compared to WT; *p < 0.05; *n *= 8 per genotype. No changes were detected in brain insoluble factions.

The OxyBlot kit was used to detect the levels of oxidized proteins as a measure of oxidative stress in brain and spinal cord fractions. The level of oxidized proteins in the soluble brain and spinal cord fractions (containing mostly cytosolic proteins) of TDP-43^A315T^ mice was not different from wild-type mice (**Figure 3A**). However, when assessing the insoluble fraction, containing mainly membrane and nuclear material, there was a significant 2.2-fold increase in the level of oxidized proteins in the spinal cord of TDP-43^A315T^ mice when compared to wild-type (*p* < 0.05; **Figure 3B**). There were no differences in the insoluble brain fraction between genotypes (**Figure 3B**).

### INCREASED MARKERS OF INFLAMMATION IN THE BRAIN AND SPINAL CORD OF TDP-43^A315T^ MICE

**FIGURE 4:**
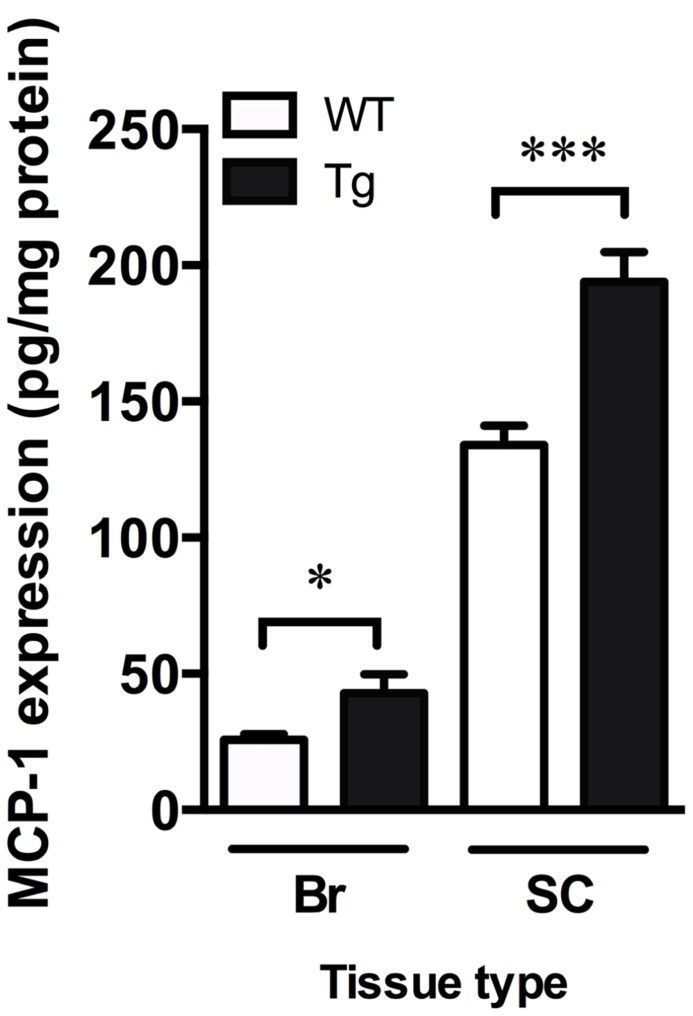
**Increased markers of inflammation in TDP-43^A315T^ mice**. MCP-1 ELISA show a significant increase in MCP-1 content in the brain (Br) and spinal cord (SC) of TDP-43^A315T^ mice (Tg) compared to wild-type (WT). ***p < 0.001; *p < 0.05. *n *= 9 per genotype for brain; *n *= 7 per genotype for spinal cord.

Monocyte chemoattractant protein-1 is involved in microglia-mediated inflammatory processes in the CNS and can be used as an indicator of increased inflammation (Deshmane et al., 2009). Levels of MCP-1, as detected by ELISA, were significantly increased in the brain and spinal cord of TDP-43^A315T^ mice by 1.7- and 1.4-fold, respectively (*p *< 0.05; **Figure 4**).

## DISCUSSION

Metal dyshomeostasis is implicated in a number of neurodegenerative diseases (Sofic et al., 1988; Connor et al., 1992; Lovell et al., 1998), but whether metals play a role in the degeneration of neurons in TDP-43 associated forms of diseases such as FTLD-U and ALS is unknown. In this study, the TDP-43^A315T^ mouse model of FTLD/ALS TDP-43 proteinopathy was used to examine potential metal changes in the CNS due to expression of a pathogenic form of mutant TDP-43. Consistent with previous studies (Wegorzewska et al., 2009), FLAG-tagged mutant TDP-43 was mainly expressed within the CNS of TDP-43^A315T^ mice resulting in the overexpression of TDP-43 in the brain and spinal cord. These animals exhibited a locomotor impairment reminiscent of ALS. The TDP-43^A315T^ mice performed poorly on the rotarod test and demonstrated a progressive loss of locomotor activity towards end-stage. This observation is consistent with those reported by Esmaeili and colleagues (2013). Full hind-limb paralysis was not evident prior to the premature death of the TDP-43^A315T^ mice.

Bulk metal analysis of the spinal cord revealed a significant increase in the levels of manganese, Cu and Zn due to expression of mutant TDP-43 in the TDP-43^A315T^ mice (**Figure 2**). These changes appear specific to the spinal cord tissue as manganese, copper and zinc were not altered in the brain, liver, or quadriceps muscle (**Figure 2**). Rotarod analysis of the TDP-43^A315T^ mice revealed a clear deficit relative to wild-type mice, but the Y-maze assessment of the TDP-43^A315T^ mice indicated expression of the mutant TDP-43 did not affect the cognitive function of these animals (**Figure 1D**). This apparent demarcation between symptoms of neuronal decline in the spinal cord compared to neuronal decline in the brain is despite the higher levels of the mutant TDP-43 expression in the brain compared to spinal cord (**Figure 1B**). Thus, the effects of mutant TDP-43 expression on metals in the spinal cord, but not the brain, may have contributed to the predominantly locomotor phenotype of the TDP-43^A315T^ mice.

The mechanisms by which mutant TDP-43 could contribute to elevated levels of these metals in the spinal cord remain to be fully elucidated, and it is not yet clear whether the altered metal content of the spinal cord represents a cause or consequence of neuronal dysfunction. The data generated for levels of oxidized proteins (**Figure 3**), however, are in part consistent with the altered metal content representing a causative event. Despite no change to levels of oxidized proteins in the soluble fraction, the abundance of oxidized proteins in the insoluble fraction was elevated in the spinal cords of TDP-43^A315T^ mice. Oxidative damage is evident in the spinal cords of ALS cases (Niebroj-Dobosz et al., 2004) as well as mutant SOD1 mouse models of the disease (Soon et al., 2011), and altered metal homeostasis has been associated with this oxidative damage. Most notably, the pro-oxidant toxic gain-of-function ascribed to mutant SOD1 in SOD1-associated cases of ALS is proposed to be the result of altered metallation of SOD1 (Beckman et al., 2001). The normal anti-oxidant activity of SOD1 requires equimolar binding of Zn and Cu, but disruptions to this metal stoichiometry, including disruptions caused by ALS-associated SOD1 mutations (Crow et al., 1997; Roberts et al., 2007) confer toxic pro-oxidant activity to the SOD1 (Estevez et al., 1999). Significantly, altered metallation of wild-type SOD1 also confers a pro-oxidant toxic gain-of-function (Estevez et al., 1999). It is possible therefore, that the mutant-TDP-43 induced disruption of metal homeostasis detected in the spinal cords of the TDP-43^A315T^ mice contributed to a neurotoxic oxidative mechanism already proposed as a significant pathogenic event in ALS.

In contrast to oxidative damage, the data generated for levels of the inflammatory marker MCP-1 are less clear with respect to inflammation possibly contributing to the mutant TDP-43-induced phenotype of the TDP-43^A315T^ mice. Levels of MCP-1 were elevated in the spinal cords of TDP-43^A315T^ mice, and they were also elevated in the brain (**Figure 4**). Given the mice did not display an overt cognitive phenotype indicative of neuronal dysfunction in the brain, these data may suggest the inflammation present in the brains of the TDP-43^A315T^ mice represents a relatively non-specific response to the over-expression of an exogenous protein. Alternatively, these data may represent a specific consequence of mutant TDP-43 expression, a possibility consistent with evidence for increased inflammation in ALS and FTLD (Galimberti et al., 2008; Letiembre et al., 2009; Papadimitriou et al., 2010). As discussed below, the absence of an overt cognitive phenotype in the TDP-43^A315T^ mice may be due to the premature death of these animals preventing the opportunity for the manifestation of neuronal dysfunction in the brain.

The data presented in this study indicate increased metal levels in the spinal cords of the TDP-43^A315T^ mice may have contributed to their locomotor impairment, while the lack of cognitive impairment may be due to the absence of metal dyshomeostasis in the brain. However, this study only performed bulk metal analysis whereby whole tissue homogenates were analyzed for total metal levels. Region specific changes to metals or the redistribution of metals in the brain may therefore have been undetected by this methodology. Alternate analytical techniques such as laser-ablation ICP-MS which allows for spatial resolution of metal concentrations within tissues (Hare et al., 2012) or liquid chromatography ICP-MS which allows resolution of metalloproteins prior to metal quantitation (Lothian et al., 2013) may therefore be required before excluding the possibility that expression of mutant TDP-43 caused relatively subtle changes to metals within the brains of the TDP-43^A315T^ mice. In addition, it must be noted that the TDP-43^A315T^ mouse model has recently been reported to die prematurely from gastrointestinal complications before the development of full ALS- and FTLD-like symptoms (Guo et al., 2012; Esmaeili and colleagues, 2013). Although we did not study these gastrointestinal complications, we did find that the TDP-43^A315T^ mice died suddenly before the presence of full hind-limb paralysis. This raises the possibility that if these mice had not died from their gastrointestinal complications they may have gone on to exhibit symptoms of cognitive decline. Thus, the non-CNS related premature death of the TDP-43^A315T^ mice may have been the only factor that limited the detection of potential alterations to brain metal homeostasis and/or cognitive impairment, especially if FTLD-like symptoms develop much later than ALS-like symptoms.

Overall, this preliminary study is the first to report altered metal content in the spinal cord of the TDP-43^A315T^ mouse model of FTLD and ALS. The increase in metal content is associated with increased oxidative stress and inflammation in this tissue. The mechanism by which mutant TDP-43 can alter metal levels and the effect of these changes on the oxidative and inflammatory status of the CNS remains to be elucidated, and an analysis of these changes to spinal cord metal levels is needed to address their temporal relationship with the progressive phenotype of the TDP-43^A315T^ mice. Nonetheless, the data from this study provide evidence to support the role of metal dyshomeostasis in neurodegenerative diseases, including TDP-43 proteinopathies.

## Conflict of Interest Statement

The authors declare that the research was conducted in the absence of any commercial or financial relationships that could be construed as a potential conflict of interest.
